# The prevalence of depression and associated factors in Ethiopia: findings from the National Health Survey

**DOI:** 10.1186/1752-4458-6-23

**Published:** 2012-10-25

**Authors:** Solomon Hailemariam, Fasil Tessema, Mekonen Asefa, Henok Tadesse, Girma Tenkolu

**Affiliations:** 1 School of health science, Dilla University, Dilla, Ethiopia; 2 Department of epidemiology and Biostatistics, Jimma University, Jimma, Ethiopia

## Abstract

**Background:**

Integrating mental health into primarily health care and studying risk for mental health particularly depression needs assessment of different factors including those that impede diagnosis and treatment of mental disorders. But so far the numbers of literature for local context to analyze risk factors for depression and its treatment are scare. The objective of this study was to assess risk factors and health service attendance for depression among adults, in Ethiopia.

**Methods:**

For this analysis, data from the Ethiopian National health survey was used. The Ethiopian national health survey studied 4,925 adults aged 18 years and older to obtain among other things, data on depression episodes, socio-demographic, chronic diseases, life style factors and treatment receiving for depression episodes in the past twelve months using questionnaire from world health organization (WHO). Prevalence of Depression in respondents based on ICD-10 criteria was estimated and logistic regression analysis was used to identify risk factors for depression and treatment receiving.

**Results:**

The prevalence of depressive episode was 9.1% (95% CI: 8.39-9.90). In a Univariate analysis, residence, age, marital status, educational status, number of diagnosed chronic non communicable diseases (heart diseases, diabetic mellitus and arthritis) and alcohol drinking status were associated with depression. After full adjustment for possible confounding, odds ratios for depression were significantly higher only for older age, divorced and widowed, number of diagnosed chronic non communicable diseases and alcohol drinking status. The proportion of attending health service among those with depression episodes was 22.9%. After full control for all socio-demographic variables the only predictor variable was educational status, being in grade 5–8 had a higher odds (OR=2.6, 95% CI: 1.23-5.43) and 9–12 grade (OR=1.8 95% CI: 1.45-6.12) of attending service for depressive episodes.

**Conclusions:**

Age, marital status, number of diagnosed chronic non communicable diseases and alcohol consumption were the most important risk factors for depressive episodes. Generally there was lower use of health service for depressive episodes and low educational status was found to be barriers for service use. There is a need to formulate policy for mental health and training of primary health care workers in mental health to early identify and treat cases with depression episodes, so as to decrease prevalence of depression episodes and to improve accessibility of service use.

## Background

Poor mental health can result in poorer outcomes associated with other diseases such as cancer, HIV/AIDS, diabetes and cardiovascular disease [1,2]. Depression is the fourth most important contributor to the global burden of disease and comprises in year 2000 4.4% of the total Disability Adjusted Life Years [3,4].

In three European countries (Iceland, Norway and Switzerland), the point prevalence of uni-polar depression is 1.9% for males and 3.2% for females [5]. The consequences of psychiatric morbidity especially depression, if it is not identified and treated, can be severe [3]. These include suicide, loss of jobs and relationships, loss of productivity and deterioration in physical health including higher risk of myocardial infarction [1,4,6].

Studies from both middle and high income countries [7-11] have shown that depression is common among older people than adolescence. Studies have shown many risk factors for depression: Physical and functional health problems, loneliness and financial worries [10,12]; marital state [11,13], years of formal education, employment status [13]; Socio-demographic factors such as gender and age [7,11]; disability, poor social support and cognitive function [14]; non communicable disease such as diabetic, hypertension, stroke, lung disease and arthritis [9,11]; history of self-harm, family history of psychiatric problems and substance use [15]; exposure to violence and crime, and acculturation stress [10].

The global neglect of mental health is well-documented [4,14]. In most countries, mental health issues are neglected within health care policy and planning and only limited resources are allocated to mental health services [3].

In Ethiopia mental Health has been one of the most disadvantaged health programs, both in terms of facilities and trained manpower, however, during the last decade; encouraging efforts have been taken to expand services throughout the country. Recognizing the need to scale up mental health services, the Ethiopian Government has shown political commitment to the successful implementation of the mental health GAP action (mhGAP) programme supported by the Foundation d’Harcourt and the European Commission. Regarding human resource there are 36 psychiatrist and more than 400 psychiatry nurses [16].

Studies conducted in Ethiopia showed the 12 month’s prevalence of depression to be: 4.4% [17], and 4.8% [18] among women. Moreover, the life time prevalence of depression in general population was reported 2.2% [19], minor depressive disorder 20.5% [20], depressive episodes 2.7% and recurrent depressive episodes 0.2% [21]. In a case cohort study conducted to investigate outcome of major depression indicated that age and sex adjusted mortality ratio is 3.55 and the incidence of disability is also high among persistently depressed group [22]. As regards mental disorders studied in Ethiopia, the prevalence of dissociative disorders, mood disorders, somatoform disorders and anxiety disorders is 31.8% [23], neurotic and somatoform disorders and affective disorders 18.3 [24]. Two large scale studies estimated the prevalence of mental distress using the same measurement (self-reporting questionnaire) showed 11.7% in Addis Ababa [25], and 17% in Butajira [26]. The occurrence relation of help seeking behaviour and depression in Ethiopia is not well understood, except one cohort studied and found that 22.2% of individuals with persistent depression sought help in the past 3 months before the follow up time [22].

Taking this complex nature of the problem and disease burden, this study tries to identify risk factors and health service attendance for depression. It is our belief that the findings of this study will give relevant information to help efforts in integrating mental health into primarily health care and the effort in scaling up services for mental disorders in Ethiopia.

## Methods

### Study area

The study was conducted in Ethiopia. Ethiopia is a country with an approximate area of 1,104 sq. Km with population in 2008 of about 85 million and the sex ratio (men per hundred women) is 99. The country has 53 psychiatric outpatient facilities, 6 inpatient facilities and one mental hospital. There is only one residential facility in the country for chronically mentally ill and several other residential facilities, which have served mentally ill clients among with their beneficiaries. There are 0.35 mental hospital beds and 0.14 community psychiatric inpatient beds per 100,000populations. In terms of training, 2% and 3% of the training medical doctors and nurses respectively are devoted to mental health. The Ethiopian National Health Survey (ENHS) was conducted in nine regions, including Addis Ababa, Dire Dawa, Harari, Amhara, Oromia, Southern Nation, Nationalities and Peoples Region, Tigray, Gambela, and Benshangul.

### Study design

This was a cross-sectional study and used the data from the 2003 ENHS collected by Jimma University in collaboration with WHO.

### Source population

All Ethiopian population aged 18 and above, including Addis Ababa, Dire Dawa, Harari, Amhara, Oromia, Southern Nation, Nationalities and Peoples Region, Tigray, Gambela, and Benshangul.

### Sample size and sampling technique

As described elsewhere [27], a multistage stratified sample survey was conducted. The stratification variables used were regions and residence. Following the administrative levels in each setting the following selection stages were incorporated: Woreda (District), Kebele, and Household and individual. In each sampling stage probability methods were employed. From selected house-holds adults 18 years and over were selected using Kish table. From 5,131 selected house-holds 4990 adults 18 years and over were selected and 4,936 were interviewed. For this analysis, due to some missing values, the final sample size was 4925 (98.7%) respondents.

### Data collection

All respondents used in the analysis were interviewed with the standardized world health survey questionnaire developed by WHO. The questionnaire has been developed in multiple languages using cognitive interviews and cultural applicability test and utilizing novel psychometric techniques for cross-population comparability. All surveys were implemented using face-to-face interviews. A detail of the data collection process is described elsewhere [27].

### Dependent variables

#### Depression and treatment received

The diagnosis of depression episodes was based on the International Classification of Diseases tenth revision (ICD-10) diagnostic criteria for research for depressive episodes [28] and was derived from an algorithm that took into account respondents reporting symptoms of depression during the past 12 months. The individual questions used to assess these symptoms were based on the World Mental Health Survey version of the Composite International Diagnostic Interview [29]. This was validated in seven countries by WHO 2003, using diagnostic item probability study and reported to perform well [30].

Questions used to identify depression episodes during the survey were:

1. Have you had a period lasting several days when you felt sad, empty or depressed?

2. Have you had a period lasting several days when you lost interest in most things you usually enjoy?

3. Have you had a period lasting several days when have been feeling your energy decreased?

4. Was this period (of sadness/loss of interest/low energy) more than 2 weeks?

5. Was this period (of sadness/loss of interest/low energy) most of the day, nearly every day?

6. During this period did you lose your appetite?

7. During this period did you notice any slowing down in your thinking?

Respondents will be coded in to two categories following ICD-10 minimum diagnostic criteria for research for depressive episodes [28].

I. Depression episodes with at least two of the following 3 symptoms:

1. Depressed, sad, empty present for most of the day and almost every day and sustained for more than two weeks.

2. Loss of interest or pleasure in activities that is normally pleasurable present most of the day and almost every day and Sustained for more than two weeks.

3. Feeling energy decreased that present most of the day and almost every day sustained for more than two weeks.

And additional one or more of the following symptoms:

1. Loss in appetite (during feeling energy decreased/sadness/loss of interest)

2. Noticing in slowing down in thinking (during feeling energy decreased/sadness/loss of interest).

II. No depression episodes for respondents that don’t full fill the above criteria

Respondents were asked whether they have attended health service for depression in the past 12 months.

### Predictor variables

1. *Socio-demographic factors*: During the interview, demographic data were obtained on age, sex, residence, educational level, marital status, and income.

2. *Alcohol Use*: Alcohol use was assessed using the question, ‘How often do you drink anything containing alcohol?’ and frequency and quantity were measured. Responses were coded into four categories following WHO recommends: life time abstainer, non-heavy drinker, 1–2 days with 5 or more standard drinks per week (infrequent heavy drinkers) and 3 or more days with 5 or more standard drinks per week (frequent heavy drinkers).

3. *Self-reported Chronic Illnesses*: Participants were asked whether or not they were having received diagnosis of the condition (e.g. diabetes, arthritis, heart disease).

### Data analysis

All analyses were conducted using Statistical Package for Social Sciences version 16. Descriptive statistics was used to describe the study sample. The results were then expressed as percentage, and respondents was aggregated into two groups consisting of those with depression episodes and no depression episodes. Respondents were also aggregated into two groups consisting of those attend and not attend health service for depression. Chi square trend analysis was also carried out for the depression episodes with respect to age, educational status and exposure to chronic non communicable diseases. Associations between risk factors and depression episodes were analyzed using multiple logistic regressions. Odds Ratios (OR) was generated for each variable and the independence of any association was initially controlled for sex and age, then additionally by entering all variables into the model. The magnitude of the association between the different variables in relation to depression and treatment was measured using odds ratios (OR) and 95% confidence interval (CI) and P-values below 0.05 was considered statistically significant.

### Ethical clearance

The ENHS was cleared by Jimma Ethical Committee. Before the commencement of analysis of the survey data, letter of permission to use the data was obtained from the principal investigators of the national survey, Jimma University.

## Results

### Socio-demographic characteristics

The sociodemographic characteristics of the respondents are shown in Table 1. From 5131 selected households, 4925 (84.9%) from rural and (15.1%) from urban individuals were responded for depression episodes questions. The socio-demographic data showed that 45.7% of the subjects were found between 18 and 30 years, with mean age of 36.1 (SD= 15.4) and 51.6% were women and 48.4% men. The majority of the respondents were currently married (68.0%). Most of the study subjects were illiterate (52.6%) and only (3.7%) had higher education. About (41.5%) of the population was unemployed and the majority (93.2%) had a monthly income below 500 birr.

**Table 1:** Socio demographic characteristics of respondents by residential area, Ethiopia 2003

	**Residence**					
	**Urban**		**Rural**		**Total**	
	**n**	**%**	**n**	**%**	**n**	**%**
Sex						
Male	317	42.5	2066	49.4	2383	48.4
Female	429	57.5	2113	50.6	2542	51.6
Total	746	100.0	4179	100.0	4925	100.0
Age group						
18-30	359	48.1	1894	45.3	2253	45.7
31-34	175	23.5	1113	26.6	1288	26.2
45-54	94	12.6	520	12.4	614	12.5
54-64	57	7.6	360	8.6	417	8.5
65-74	44	5.9	197	4.7	241	4.9
75+	17	2.3	95	2.3	112	2.3
Total	746	100.0	4179	100.0	4925	100.0
Marital status						
Never married	240	32.2	623	14.9	863	17.5
Currently married	363	48.7	2988	71.5	3351	68.0
Single	18	2.4	70	1.7	88	1.8
Divorced	44	5.9	190	4.5	234	4.8
Widowed	81	10.9	308	7.4	389	7.9
Total	746	100.0	4179	100.0	4925	100.0
Educational status						
Illiterate	167	23.3	2257	58.1	2424	52.6
1-4 grade	85	11.9	598	15.4	683	14.8
5-8 grade	132	18.4	617	15.9	749	16.3
9-12 grade	227	31.7	352	9.1	579	12.6
Higher	106	14.8	64	1.6	170	3.7
Total	717	100.0	3888	100.0	4605	100.0
Current job						
Employed	327	44.6	2531	61.0	2858	58.5
Unemployed	406	55.4	1618	39.0	2024	41.5
Total	733	100.0	4149	100.0	4882	100.0
Income group						
≤ 500 birr	590	82.9	3924	95.0	4514	93.2
501-999 birr	85	11.9	170	4.1	255	5.3
≥ 1000 birr	37	5.2	37	0.9	74	1.5
Total	712	100.0	4131	100.0	4843	100.0

### Age- and gender-specific prevalence proportions of depressive episodes

According to the ICD-10, 449 adults with symptoms of depressive episodes in the last 12 month before survey were identified with over all prevalence proportion of 9.1% (95% CI 8.31–9.90). While 8.7% (95% CI 7.66–9.83) of the males suffered from depression episodes, the proportion among females was 9.5% (95% CI 8.36–10.64).

Table 2 contains Univariate OR within categories of the various independent variables and in further analysis adjusted OR first for age and sex and then for all variables were shown in Table 3.

**Table 2:** Univariate association of different variables with depression episodes, Ethiopia 2003

**Factors**	**Respondents n**	**Depression episodes n (%)**	**OR (95% CI)**	**P-value**
Residence				
Rural	4179	397 (9.5)	1.4 (1.04-1.89)	0.028
Urban	746	52 (7.0)	1	
Sex				
Male	2383	207 (8.7)	1	
Female	2542	242 (9.5)	1.1 (0.91-1.34)	0.310
Marital status				
Never married	863	30 (3.5)	1	
Currently married	3351	302 (9.0)	2.8 (1.88-4.03)	<0.001
Separated	88	6 (6.8)	2.0 (0.82-5.02)	0.125
Divorced	234	34 (14.5)	4.7 (2.82-7.91)	<0.001
Widowed	389	77 (19.8)	6.8 (4.41-0.66)	<0.001
Current job				
Employed	2858	277 (9.7)	1	
Unemployed	2024	172 (8.5)	0.95 (0.71-1.15)	0.155
Income level				
≤ 500 birr	4514	410 (9.1)	1	
501–999 birr	255	29 (11.4)	1.3 (0.86-1.92)	0.221
≥ 1000 birr	74	10 (13.5)	1.6 (0.81-3.07)	0.193
Alcohol consumption				
Life time abstainer	3049	189 (6.2)	1	
Non heavy drinkers	1292	187 (14.5)	2.6 (2.07-3.17)	<0.001
Infrequent heavy drinkers	152	22 (14.5)	2.6 (1.59-4.12)	<0.001
Frequent heavy drinkers	418	51 (12.2)	2.1 (1.52-2.92)	<0.001

**Table 3:** Adjusted association of different variables with depression episodes, Ethiopia 2003

**Factors**	**OR adjusted for age & sex (95%CI)**	**OR adjusted for all variables (95%CI)**	**P-value**
Residence			
Rural	1.4 (1.06-1.96)	1.1 (0.81-1.61)	0.495
Urban	1	1	
Sex			
Male	1	1	
Female	1.3 (1.02-1.53)	0.9 (0.68-1.19)	0.458
Age group			
18–30	1	1	
31–44	1.7 (1.28-2.18)	1.1 (0.85-1.52)	0.403
45–54	2.2 (1.62-3.01)	1.3 (0.98-1.79)	0.182
55–64	3.2 (2.34-4.46)	1.4 (1.01-2.14)	0.042
65–74	4.4 (3.09-6.39)	1.8 (1.21-2.81)	0.005
75+	6.0 ( 3.78-9.65)	2.2 (1.28-3.78)	0.004
Educational status			
Illiterate	1.5 (0.87-2.78)	0.9 (0.43-1.73)	0.675
1-4 grade	0.9 (0.46-1.81)	0.6. (0.31-1.35)	0.249
5-8 grade	1.1 (0.54-2.10)	0.8 (0.39-1.62)	0.521
9-12 grade	1.2 (0.58-2.33)	1.0 (0.49-2.09)	0.969
Higher	1	1	
Marital status			
Never married	1	1	
Currently married	1.9 (1.31-2.99)	1.5 (0.95-2.33)	0.081
Separated	1.4 (0.57-3.65)	1.2 (0.45-3.15)	0.733
Divorced	3.0 (1.73-5.28)	2.0 (1.12-3.72)	0.023
Widowed	3.3 (1.79-5.64)	2.4 (1.39-4.28)	0.002
Current job			
Employed	1	1	
Unemployed	0.85 (0.68-1.07)	0.98 (0.77-1.26)	0.895
Income level			
≤ 500 birr	1	1	
501–999 birr	1.3 (0.87-1.97)	1.4 (0.89-2.10)	0.155
≥ 1000 birr	1.6 (0.81-3.22)	1.9 (0.90-3.91)	0.092
Number of diagnosed chronic diseases			
None	1	1	
One	2.8 (2.20-3.50)	2.6 (2.03-3.25)	< 0.001
2+	4.9 (3.79-6.55)	4.2 (3.18-5.57)	< 0.001
Alcohol consumption			
Life time abstainer	1	1	
Non heavy drinkers	2.3 (1.87-2.89)	2.0 (1.63-2.57)	< 0.001
Infrequent heavy drinkers	2.5 (1.54-4.09)	2.2 (1.33-3.64)	< 0.001
Frequent heavy drinkers	1.9 (1.42-2.79)	1.6 (1.13-2.28)	0.002

### Socio-demographic factor

Independent of their age and sex people from rural suffer from depressive episodes significantly more often than people from urban (OR=1.4, 95% CI 1.06–1.95). After controlling for all variables, this association is no longer significant (OR=1.1, 95% CI 0.81–1.61).

The proportion of depression episodes is higher among women (9.5%) than men although the observed difference is not statistically significant (OR=1.1, 95% CI 0.91–1.34).

Chi-square test for trend analysis showed that age is associated with the risk of depression episodes, the risk increasing with increasing age (Table 4). This direct trend was statistically significant (*P* for trend <0.0001). controlling for potential confounder variables, age was a risk factor for developing depression episodes: 31–44 years of age group had a higher odds of (OR=1.7, 95% CI 1.28–2.18), 45–54 years of age (OR=2.2, 95% CI 1.62–3.01), 55 –64 years of age group (OR=3.2, 95% CI 2.34–4.46), 65 –74 years of age group (OR=4.4, 95% CI 3.09–6.39) and those 75 years of age and older (OR=6.0, 95% CI 3.78–9.65) developing depression episodes compared to those in age between 18 and 30 years. But after fully adjusted for all variables those 55–64 years old had a higher risk (OR=1.4, 95% CI 1.01–2.14), 65–74 years old (OR=1.8, 95% CI 1.21–2.81) and 75 years and older had a risk (OR=2.2, 95% CI 1.28–3.78) of developing depression episodes which was statistically significant association.

**Table 4:** Chi-square test for trend analysis of age with depression episodes, Ethiopia 2003

**Age group**	**Depression episodes**		**Total n (%)**
	**Yes**	**No**	
18-30	125 (5.5%)	2128 (94.5%)	2253 (100)
31-44	113 (8.8%)	1175 (91.2%)	1288 (100)
45-54	69 (11.2%)	545 (88.8%)	614 (100)
55-64	65 (15.6%)	352 (84.4%)	417 (100)
65-74	49 (20.3%)	192 (79.7%)	241 (100)
75^+^	28 (25%)	84 (75%)	112 (100)
Total	449 (9.1%)	4476 (90.9%)	4925 (100)
**Trend Analysis**			
Chi-square test	Value	df	P-value
Pearson chi-square	129.92	5	< 0.001
Chi-square for trend	127.92	1	< 0.001

Education is associated with the risk of depression episodes, the risk decreasing with increasing educational attainment (Table 5), this inverse trend was statistically significant (*P* for trend <0.0001). After controlling for age, gender and all variables the association was no longer significant.

**Table 5:** Chi-square test for trend analysis of educational status with depression episodes, Ethiopia 2003

**Educational status**	**Depression episodes**		**Total n (%)**
	**Yes**	**No**	
Illiterate	305 (12.6%)	2119 (87.4%)	2424 (100)
1-4 grade	44 (6.4%)	639 (93.6%)	683 (100)
5-8grade	49 (6.5%)	700 (93.5%)	749 (100)
9-12 grade	40 (9.6%)	539 (93.1%)	579 (100)
Higher	11 (6.5%)	159 (93.5%)	170 (100)
Total	449 (9.8%)	4156 (90.2%)	4605 (100)
**Trend Analysis**			
Chi-square test	Value	df	P-value
Pearson chi-square	46.74	4	< 0.001
Chi-square for trend	32.79	1	< 0.001

With regard to marital status, a greater number of depressive subjects are found in the group of widowed respondents (19.8%), as well as in the divorce group (14.5%), and in the married group (9.0%), and especially in the single group (6.8%). In Univariate and OR adjusted for age and sex being married (OR=1.9, 95% CI 1.31–2.99), divorced (OR=3.0, 95% CI 1.73–5.28) and widowed group (OR=3.3, 95% CI 1.97–5.64) was found to be risk factors for depression episodes. But after adjusting for all other variables the most important risk factor for depression episodes were those divorced and widowed, those divorced (OR=2.0, 95% CI 1.12–3.72), and those widowed (OR=2.4, 95% CI 1.39–4.28) had a higher risk of developing depression episodes.

Concerning employment status, the number of depressive are higher (9.7%) in employed group compared to those unemployed (8.5%) the difference is not statistically significant (OR=0.98, 95% CI 0.77–1.26). And similar finding was also found for income, after adjusting for age, sex and all other variables with prevalence of (11.4%) in those with income between 501–999 birr (OR=1.4, 95% CI 0.89–2.10) and with high prevalence of (13.5%) in those with income level ≥ 1000 birr(OR=1.9, 95% CI 0.90–3.19).

### Lifestyle factor

Here, the proportion of depression episodes are higher in the group of non heavy drinker and infrequent heavy drinker subjects (14.5%) than in the groups whose alcohol consumption is either frequent heavy drinker (12.2%) or life time abstainers (6.2%). The risk of depression is significantly higher for the three groups: for non heavy drinker (OR=2.3, 95% CI 1.87–2.89), infrequent heavy drinkers (OR=2.5, 95% CI 1.54–4.09) and for frequent heavy drinkers (OR=1.9, 95% CI 1.42–2.79) after controlling for age and gender. And in the fully adjusted model there is little modification on the risk but the association remains the same.

### Non communicable chronic diseases

Those study participant who diagnosed for non-communicable diseases (NCDs) showed a significant trend association with depression episodes (P for trend <0.0001), the risk increasing with increasing number (NCDs) (Table 6). After fully adjusting for all variables, being having a one diagnosis of chronic non communicable disease had a higher risk for presence of depression episodes (OR=2.6, 95% CI 2.03–3.25) and having two or more diagnoses of chronic non communicable diseases further increases the likelihood (OR=4.2, 95% CI 3.18–5.57) for the presence of depression episodes as compared to those with no life time diagnoses of non-communicable diseases.

**Table 6:** Chi-square test for trend analysis of CNDs with depression episodes, Ethiopia 2003

**Number of chronic non communicable diseases**	**Depression episodes**		**Total**
	**Yes**	**No**	**No (%)**
None	188 (5.5%)	3210 (94.5%)	3398 (100)
1	151 (15.7%)	812 (84.3%)	963 (100)
2^+^	110 (27.0%)	297 (73.0%)	407 (100)
Total	449 (9.4%)	4319 (90.6%)	4768 (100)
**Trend Analysis**			
Chi-square test	Value	df	P-value
Pearson chi-square	252.42	2	< 0.001
Chi-square for trend	252.15	1	<0.001

### Attending health service among respondents with depression episodes

Table 7 shows the proportions for health services utilization for depression. Among subjects with depression episodes, 22.9% reported that they visited health facilities for their problem in the past twelve months. Services utilization for depressive episodes appeared higher among urban 30.8% dwellers. Nearly twenty percent (19.8%) of females with depression episodes reported health service use as compared to 26.6% of males with depression episodes. Health facility visits ranges from minimum 17.9% for subjects age 75 years and older to maximum 27.7% for subjects age 55–64 years old and 42.9% for subjects with 5–8 grade and 18.2% for 1–4 grade and for higher education subjects. The proportion of service utilization was higher for those in employed than unemployed (24.2 Vs 20.9).

**Table 7:** Health service attendance for depression episodes by socio demographic characteristics, Ethiopia 2003

**Variables**	**Adults with Depressive episodes (n=449)**	**Service attendance n (%)**
Residence		
Rural	397	87 (21.9)
Urban	52	16 (30.8)
Sex		
Male	207	48 (23.2)
Female	242	55 (22.7)
Age group		
18-30	125	27 (21.6)
31-44	113	30 (26.5)
45-54	69	13 (18.8)
55-64	65	18 (27.7)
65-74	49	10 (20.4)
75+	28	5 (17.9)
Marital status		
Never married	30	10 (33.3)
Currently married	302	63(20.9)
Separated	6	2 (33.3)
Divorced	34	11 (32.4)
Widowed	77	17 (22.1)
Educational Status		
Illiterate	305	61 (20.0)
1-4 grade	44	8 (18.2)
5-8 grade	49	21 (42.9)
9-12 grade	40	11 (27.5)
Higher	11	2 (18.2)
Current job		
Employed	277	67 (24.2)
Unemployed	172	36 (20.9)
Income group		
≤ 500 birr	410	87 (21.2)
501-1999 birr	29	14 (48.3)
≥ 1000 birr	10	2 (10.8)
Total	449	103 (22.9)

### Socio-demographic predictors attending health service

A multivariate logistic regression analysis was performed for unadjusted OR and then adjusted OR entering all socio-demographic factors as covariates (Table 8).

**Table 8:** Socio-demographic predictors of health service attendance for depression episodes, Ethiopia 2003

**Variables**	**Un adjusted OR (95%CI)**	**p-value**	**Fully adjusted OR (95%CI)**	**p-value**
Residence				
Rural	0.63 (0.34-1.19)	0.156	0.93 (0.45-1.91)	0.837
Urban	1		1	
Sex				
Male	1		1	
Female	0.89 (0.67-1.18)	0.442	0.71 ( 0.49-1.02)	0.063
Age group				
18-30	1		1	
31-44	1.48 (1.05-2.08)	0.026	1.49 (0.99-2.19)	0.053
45-54	0.98 (0.55-1.53)	0.743	0.80 (0.46-1.40)	0.440
55-64	1.89 (1.19-2.99)	0.007	1.45 (0.86-2.47)	0.164
65-74	1.50 (0.80-2.81)	0.202	0.99 (0.48-2.02)	0.973
75+	2.85 (1.43-5.69)	0.003	1.85 (0.85-3.99)	0.120
Marital status				
Never married	1		1	
Currently married	0.53 (0.24-1.18)	0.121	0.64 (0.39-1.06)	0.081
Separated	2.92 ( 1.35-6.34)	0.007	2.20 (0.95-5.19)	0.066
Divorced	2.15 (1.18-3.89)	0.012	1.70 (0.85-3.43)	0.137
Widowed	1.95 (1.16-3.28)	0.012	1.42 (0.71-2.85)	0.327
Educational status				
Illiterate	1		1	
1-4 grade	0.98 (0.39-2.01)	0.777	0.71 (0.29-1.73)	0.452
5- 8 grade	3.00 (1.69-5.64)	0.001	2.6 (1.23-5.43)	0.012
9-12 grade	2.00 (1.73-6.01)	0.025	1.8 (1.45-6.12)	0.032
Higher	0.98 (0.19-4.22)	0.882	0.76 (0.15-3.95)	0.745
Current job				
Employed	1		1	
Unemployed	0.83 (0.62-1.10)	0.192	0.91 (0.65-1.29)	0.607
Income group				
≤ 500 birr	0.73 (0.26-2.03)	0.551	0.69 (0.24-1.97)	0.689
501-1999 birr	1.53 (0.50-4.63)	0.454	1.68 (0.54-5.21)	0.373
≥ 1000 birr	1		1	

Service attendance for depressive episodes appeared higher among urban, although observed differences between urban and rural groups were not statistically significant (adjusted OR= 0.93, 95% CI 0.45–1.91). The Univariate analysis showed that the odds of attending health services was significantly associated with age group of 31–44 year (OR=1.48, 95% CI 1.05-2.08), 55-64 year (OR=1.89, 95% CI 1.19-2.2.99) and in those 75 year of age and older (OR=2.85, 95% CI 1.43-5.69). Even though the proportion of service attendance among female was lower but the association was not statistically significant (OR=0.71, 95% CI 0.49-1.02).

With regarded to marital status, the odds of attending health services was higher among those who were separated (OR= 2.92, 95% CI 1.35-6.34), those who are divorced (OR=2.15, 95% CI 1.18-3.89) and similarly in those widowed group the association was significant (OR=1.95, 95% CI 1.16-3.28), further this association is not significant in the full model.

The most important predictor variable found concerning educational status was that those whose educational level 5–8 grade had a higher odds of attending health services (OR=3.0, 95% CI 1.69–5.64) and those 9–12 grade (OR=2.0, 95% CI 1.73-6.01) as compared to illiterate group. This association remain the same after adjusting for all variables, (adjusted OR=2.6, 95% CI 1.23–5.43), and (OR= 1.8, 95% CI 1.45–6.12) for 5–8 grade and 9–12 grade respectively.

No independent significance difference observed in attending health services by employment status and income level, although those unemployed (OR=0.91, 95% CI 0.65–1.29) and income level ≤ 500 birr (OR=0.69, 95% CI 0.24–1.96) were less likely to attend health services as compared to those employed and income level ≥ 1000 birr respectively.

## Discussion

Different literature, cited elsewhere, evidenced that the depression related factors are complex and inter woven. Nevertheless, this study has come up with the prevalence and associated risk factors of depression in Ethiopia using large scale data, the 2003 ENHS. The prevalence of depression episodes found in this study (9.1%) was higher than other studies conducted in Ethiopia 4.4% among women [17], 4.8% [18] among women and 2.2% [19] in general population. The possible explanation for higher prevalence of depression episodes in our study is might be due to the methodological issues, the measurement tool used by other studies conducted in Ethiopia use the CIDI of the Amharic version which is different from the World Mental Health CIDI which has high sensitivity and this may lead to the high prevalence of depression observed in our study, other studies in Ethiopia showed still higher prevalence of minor depression disorders 20.5% [20] and 26% subjects found to have major depression at follow up in cohort study [22]. However, direct comparison with other studies was difficult due to the varying operationalization of depression episodes and the heterogeneous age groups, but the present finding of prevalence of depression episodes is within the range of what WHO reported, that within countries, the overall one-year prevalence of depression ranges from 5% to 20%. Variability in prevalence across countries might be due to cross-cultural limitations of diagnostic tools and reporting biases, differences in socio economic environments. Prevalence estimates also are likely to be influenced by stigma and discrimination [6,31]. Numerous community and facility-based studies indicate that depression rates are higher among women compared to men [4,11,32-34]. Although gender difference on depression episode is still controversial, the absence of significance difference of depression among gender is consistent with other findings from Ethiopia [19,20,35]. However, further investigation is recommended to investigate depression episodes differences taking in to account the time frame of study for both sexes.

Concerning the significantly higher odds ratio for age older than 54 years on depression episodes is consistent with other findings [8,13], reported significantly higher odds of having depression episodes among age older than 54 years. The finding has been confirmed by the results from Ethiopia [19,20,35].

In agreement to some other studies from Ethiopia, the current study has shown the significantly higher risk of depression episodes in widowed and divorced subjects [17,19,20,35]. The finding implies the unstable marital relationship and the loss of partner increases the risk of having depression episodes. Moreover, widowed subjects might have stress when one loss the beloved one, according to stress theory [4].

The most important predictors variable found in this study was the number of chronic non communicable disease which is supporting the findings of one meta analysis [8] and two other studies [13,36],that found number of diagnosed chronic non communicable disease (heart disease, arthritis and diabetic) were risk factors of depression episodes. In addition studies showed depression is the most common consequences of physical health problems. For example, in a large-scale national community survey, 52% of people with cardiovascular disease displayed symptoms of depression and among these, 30% met the criteria for a major depressive episode. Diabetes and Heart disease frequently coexist with depression and they have also a high mortality rate than the general population [4]. Although the diagnosis of NCDs is based on self report, this finding indicates the need for further research using objective diagnostic method for NCDs.

Another important predictor variable for depression episodes in our study is alcohol consumption. Where, heavy drinker, infrequent heavy drinkers and frequent heavy drinkers are significantly associated with depression episodes. This is inconsistent with other finding [11] that showed no independent difference between alcohol and depression episodes. This may be due to the difference in study subjects; however, further investigation recommended.

The level of health facility visit for depression episodes showed lower in the current study. Because the study relies on interview and it is possible that during the initial stages the signs and symptoms of depression is often quite non-specific making it difficult for the sufferers to realize that they are in need of help [37]. Another study conducted in Ethiopia showed that 22.2% had used any kind of health service for persistent depression in the past 3 months prior to the study conducted [22]. The substantial service utilization gap between the socio-demographic variables studied implies the need for further research that may be barrier for help seeking like equal access to health facilities.

The finding of independent significance association observed for health facility visit by educational status is in line with the finding from other country [38] who reported students generally have higher odds of visiting health facilities than people who have completed their education or than illiterate. Particularly taking the lower proportions of health facility visit for depression episodes in those who are in higher education and primary school the findings of this study has the implication for further research, what cultural, environmental, attitudes play as a barrier for educated and primary school people and taking appropriate measure.

One of the strengths of this study was the large representative sample of comprising of all people from all regions and social classes in Ethiopia. So this study demonstrated a possible representative prevalence of depression episodes and factors associated with it across the country.

Collecting self-reports of data on risk factors and health service utilization for depression will make it easier to empirically assess epidemiologic associations between various factors and depression, especially in countries like Ethiopia, where objective health data are lacking.

The present findings are discussed and must be considered within the following limitations. First, the identification of individuals with depression episodes and health service attendance by interviewing in the past 12 months, which is subject to recall bias. Secondly, The ENHS was a cross-sectional study and didn’t include questions on onset and duration of illness and details of health care use such as number and timing of contact with health care services, as such this study cannot address the adequacy, type and delay of treatment for depression. Thirdly, although the Amharic version of CIDI showed good reliability, feasibility and acceptance in different cultural backgrounds, the major problem is inappropriateness for the out-patient clients and its lengthy questions [39]. Validity of the CIDI is also problem because of strict diagnostic rule. Moreover there is no gold standard diagnosis for depression even the clinical diagnoses. So the prevalence of depression episodes mentioned here must be considered with this limitation.

## Conclusions

The study revealed a relatively high prevalence of depression episodes. Older age, marital status, number of diagnosed chronic non communicable diseases and alcohol consumption found to be the most important risk factors for depressive episodes. This has been worsening by the lower health facility visit for depression episodes. The study findings indicate the importance of strategies to fully integrate mental health services in to primary health care in order to decrease the prevalence of depression. What is more? It needs to build the capacity of primary health care workers to early identify and manage depression episodes cases. Depression can be treated at primary health care or at community settings with locally available cost effective interventions. In addition, the most important barriers observed for health service use is low educational status, which indicate the need to allocate public funding for the provision of mental health services and treatment, taking in to consideration the risk factors identified here.

## Competing interests

The authors declare that they have no competing interests.

## Authors’ contributions

SH contributed to generate topic, writing proposal, analyses and development of the manuscript. FT contributed to design of the ENHS, data collection, give comments on the proposal and manuscript. And MA contributed to design of the ENHS and data collection. HT contributes critically review of the manuscript and process the publication. GT critically reviewed the manuscript. All authors read and approved the final manuscript.
